# Heavy metal contamination from fuel station run-off and carwash wastewater: An assessment of ecological risk and experimental treatment

**DOI:** 10.1016/j.heliyon.2024.e29167

**Published:** 2024-04-03

**Authors:** Emmanuel Leekplah Cuput, Lawson Mensah, Ethel Bentil, Victoria Amponsah, Bright Kwaku Agbekey

**Affiliations:** Department of Environmental Science, Faculty of Biosciences, College of Science, Kwame Nkrumah University of Science and Technology, Kumasi, Ghana

**Keywords:** Carwash wastewater, Fuel station run-off, Heavy metal, Ecological risk assessment, Charcoal-augmented sand filter

## Abstract

Fuel station run-off (FSR) and carwash wastewater (CWW) are potential sources of heavy metals contamination in surface waters. High levels of heavy metals can have dire consequences on the ecosystem in receiving waterbodies. Ghanaians demand (and supply) of fuel and vehicle cleaning services has increased sharply with population and economic growth and the trend will continue. In this study, a microwave plasma atomic emission spectrometer was used to analyze the levels of Cr, Cu, Ni, Pb, and Zn in CWW and FSR from ten fuel stations and ten carwash facilities in the Oforikrom and Ejisu Municipalities in the Ashanti Region of Ghana. An experimental sand filter bed augmented with charcoal was used to treat the CWW and FSR. The efficacy of the treatment and the potential ecological risk posed by the untreated CWW and FSR were assessed using single- (contamination factor, CF) and multi-element ecological risk indicators (potential ecological risk index, PERI). The findings showed that CWW contained 0.07 mg/L Cr, 0.20 mg/L Cu, 0.02 mg/L Ni, 1.16 mg/L Pb, and 0.58 mg/L of Zn, while, FSR contained 0.05 mg/L Cr, 0.09 mg/L Cu, 0.17 mg/L Ni, 0.31 mg/L Pb, and 0.18 mg/L Zn. Copper levels in CWW and FSR were positively correlated (0.8), suggesting similar sources of contamination. CF revealed that Cr, Cu, Ni, and Zn in CWW and FSR posed low to medium risk, while Pb posed high risk. The PERI also ranked Pb in all samples as extreme pollution, and Ni as severe pollution in FSR. The charcoal augmented sand filter effectively removed Pb (96%), Cu (61%), and Zn (79%) in both CWW and FSR. Therefore, Ghana EPA and Department of Urban Planning policies should include the construction of a charcoal-augmented sand filtration system at FS and CW facilities to intercept and treat wastewater and run-off before discharge.

## 1 Introduction

Heavy metal pollution of surface and groundwater from anthropogenic activities is a major problem worldwide. Mining is a key human activity which contributes to the heavy-metal pollution of surface and groundwater sources and soil in Ghana [[1], [2], [3]]; the processing of crude oil into petroleum products in Nigeria has also been linked to groundwater contamination with lead [4], whilst and the discharge of wastewater from domestic, industrial, and agricultural settings as well as atmospheric deposition were suggested as the cause of heavy metals contamination in northern Chinese soil and sediments [5]. Although other anthropogenic sources of heavy metals into the environment are known, the scale and frequency of these activities are sometimes limited. In recent times in Ghana, the number of fuel stations (FSs) and carwash bays (CWBs) have increased rapidly and continue to rise as a result of Ghana's discovery of commercial quantities of crude oil in 2007, and the subsequent commissioning of oil rigs in 2010 [6].

Fuel stations (FSs) and carwash (CW) facilities are pivotal in supplying the energy needed to run the private and public transport sectors and providing vehicle cleaning services to the public. The country has benefited significantly from revenue and income generation through the sales of crude oil and petroleum products and its associated taxes, the direct and indirect creation of employment opportunities for the labour force in this sector, and the delivery of primary energy to the economy for domestic consumption [7]. Nationally, 80% of the energy mix is petroleum [7] and it is transacted predominantly at fuel stations up and down the country. The oil sector contributes between 0.9% and 1.4% annually to the nation's gross domestic product [7,8] and employs 4147 personnel in the upstream petroleum sector [9]. Despite the multitude of interventions employed by Ghana's energy industry to deliver innovative, high-quality, and clean energy services, natural gas and petrol remain the two most reliable energy sources [10]. Hence, fuel service providers continue to build more FSs [7] as fast-growing urban populations remain heavily reliant on fuel-powered automobiles. FSs have become not only the most significant businesses in the oil delivery system but also a part of the road infrastructure and architecture of cities and towns [11]. Similarly, CWBs continue to expand as the number of car owners increases together with the growing need for car washing services [12]. These nexuses have allowed for the continuous development of FSs and CWBs throughout the country.

Despite the enormous contribution from this sector to the economy, there are several environmental, health and safety concerns arising from the activities at the FSs and CW premises [13,14]. Fuel station run-off (FSR) may gather petroleum hydrocarbons and heavy metals such as lead and nickel from leaded fuels (gasoline) spills [15], zinc and cadmium from the vehicle tires and galvanized parts, other chemicals in lubricant oil and grease [16], as well as suspended particulates, and deposit them into water bodies in the environment [17]. Carwash wastewater (CWW) may also contain a variety of chemical substances emanating from washing chemicals and detergents, worn brake discs and pads, mud from untarred roads and dirt from transported passengers and cargo, which may contribute to the chemical oxygen demand, turbidity, total dissolved solids, and total suspended solids, surfactants, oils and greases, and heavy metals [18] in the wastewater.

The risk posed to human health by the high concentrations of heavy metals in water resources are observed in Nigeria [4,19], South Africa [20], Pakistan [21], Thailand [22] and in Ghana [23]. Heavy metals also pose serious ecological risks when assimilated into plants and animals [24]. Heavy metals are defined as naturally occurring metals with atomic number greater than 20 and an elemental density above 5 g/cm^3^, and are among the major contaminants emitted by automobiles [25]. The environmental and health impact of heavy metals in water resources has been well-documented. For instance Ref. [26], assessed the ecological risk and source apportionment of heavy metals in a contaminated river in Taiwan. Lead in drinking water is a persistent health hazard that affects learning and intellectual capabilities in both adults and children [27], while environmental lead concentration has been linked to accumulation in tissues and bones [28]. Copper is associated with phytotoxicity through the overproduction of reactive oxygen species and damage to carbohydrates, lipids, proteins, and DNA [29]. Zinc may damage soils, groundwater, and crops [30]. The primary impacts of chromium exposure include respiratory, gastrointestinal, immunological, haematological, reproductive, and developmental effects [31]. Consequently, receiving waterbodies such as rivers, which are unarguably the most essential water resources [32], may be impacted by the heavy metals in CWW and FSR, posing a threat to human health and the environment. For example, contamination of surface and groundwater resources with heavy metals in Nigeria through the exploitation of crude oil, rendered about half of water resources unfit for human consumption [4], and lead concentrations in groundwater was found to be above the allowable limits set by the Standard Organization of Nigeria and WHO [4]. Hence, the removal of heavy metals from contaminated wastewater is essential in protecting ecosystems and human health.

In Ghana, fuel stations and carwash forecourts are typically designed to slope for drainage from the facility into the adjacent gutters to avoid flooding during the rainy season. These facilities often lack adequate infrastructure to intercept and treat run-off and wastewater before discharge into the environment. Furthermore, although it is common knowledge that the majority of wastewater in Ghana is untreated and discharged into nearby water bodies [33] a great deal of previous research into wastewater treatment has focused on the centralized treatment aspect, with little emphasis on pre-treatment at sources where the wastewater is generated [30]. The choice of treatment technology to effectively remove contaminants from wastewater can depend on the cost of operation, the environmental impact of the selected treatment method, chemicals used, removal efficiency, the intended outcome of the treatment, and economic feasibility [34]. Augmented filtration systems are an example of a hybrid treatment method that combines the conventional filtration techniques with additional components which enhances the removal efficiency of heavy metals [35]. Unvegetated low-impact development technologies, which can include a sand filter [36] can reduce heavy metals in CWW. In addition [37], also reported that applied sand is a good adsorbent for heavy metals in wastewater. A substantial amount of work has been devoted to investigating water usage in CW facilities [12]. determined the average daily volume of water used and pollution loads at CWBs in the Kumasi metropolis [38]. assessed the pollution and potential ecological risk of dust from fuel filling stations.

Notwithstanding, data on the contribution of FSR and CWW to heavy metal pollution in Ghanaian rivers is lacking. The degree of ecological risk posed by lead, copper, zinc, nickel, and chromium from CWW and FSR has never been determined, and where concentrations have been analyzed, ecological risk assessment has not received the same level of attention compared to that from the mining and other industrial sectors in water, soil, and sediments [26]. For example [39], assessed heavy-metal contamination in water by determining the contamination factor and potential ecological risk factor from municipal solid waste composting plants. It is therefore important to conduct ecological risk assessment on FSR and CWW in the Ghanaian context. Ecological risk assessment of heavy metals is often done based on the concentration of individual contaminants or on a combination of heavy metals [36]. Relying solely on single-element indices can be inadequate for assessing contamination and associated risks. Multi-element measures, such as the modified degree of contamination (mCd) and the potential ecological risk index (PERI), are employed to quantify overall synergistic ecological risk [26].

In this study, the concentrations of five heavy metals (Cr, Cu, Ni, Pb and Zn) in run-off and wastewater discharged from FS and CW facilities are determined. These heavy metals were selected based on their prevalence in fuel station dust samples in the study area [24]. The single and multi-element ecological risks posed to the aquatic species in receiving water were assessed; and the efficiency of sand filter augmented with charcoal in the treatment of the FSR and CWW was evaluated.

## 2 Materials and methods

### 2.1 Study area

**Fig. 1:**
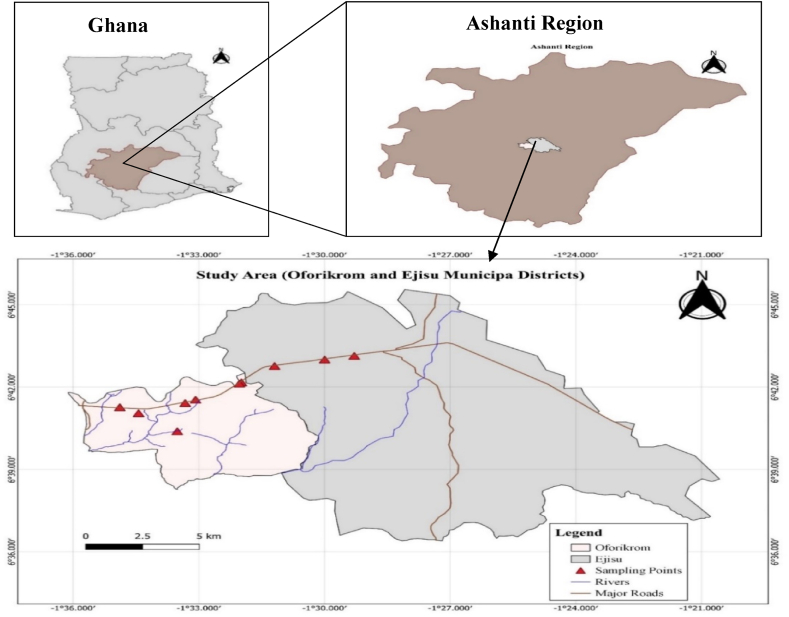
Map of the study area and the sampling locations.

The study was conducted in the Oforikrom and Ejisu municipalities surrounding the Kumasi Metropolitan [40] along the Kumasi-Accra highway (Fig. 1). Kumasi has experienced a rise in economic activities since the improved construction of the Kumasi-Accra highway [40]. Economic activities in Kumasi have increased traffic and the construction of many fuel stations and carwash facilities, especially along this road. The rationale for selecting this study area is the high density of FSs and CW premises which may have a synergistic impact on the heavy-metal contamination of streams and rivers in the area [35].

### 2.2 Sampling

This study deployed the Cochran [41] formula for sample size determination of FSs and CW facilities required, as shown in equation (1), [42].

Equation 1 $$n=\frac{NZ^{2}\sigma^{2}}{\left(N-1\right)e^{2}+Z\sigma^{2}}$$

where: *n* represents the required sample size, *N* is the population size (N = 42), *Z* is the Z-score corresponding to the desired confidence level (Z = 1.96 for 95% confidence level), ***σ*** is the population standard deviation (***σ*** = 12.3), *e* is the desired margin of error (*e* = 5, since the standard deviation has been determined, otherwise, *e* is a % of ***σ*** such as 5% of ***σ*** (*e* = 0.05***σ***).

$$n=\frac{NZ^{2}\sigma^{2}}{\left(N-1\right)e^{2}+Z\sigma^{2}}=\frac{\left(42\right){\left(1.96\right)}^{2}{\left(12.3\right)}^{2}}{\left(42-1\right){\left(5\right)}^{2}+\left(1.96\right){\left(12.3\right)}^{2}}\approx 19$$

A total of twenty locations were selected purposively, ten FSs and ten CW bays in the Ejisu and Oforikrom municipalities in the Ashanti region. Two sampling visits were made to each FS and CW bay to collect run-off during rainfall and wastewater samples in triplicates. The sample size in this study akin to those used in similar studies in other parts of Ghana [38,43]. Grab sample method [44,45], which is simple and economical to collect [46] was used. Sample bottles (1 L) were placed at the points where FSR and CWW reached the gutters/drainages until they were entirely full, allowing the samples to represent the properties of the run-off. After an aggregate of samples obtained from the FSs and CW bays were processed through the treatment system, three post-treatment samples were collected for analysis.

### 2.3 Experimental treatment of FS run-off and CW wastewater

**Fig. 2:**
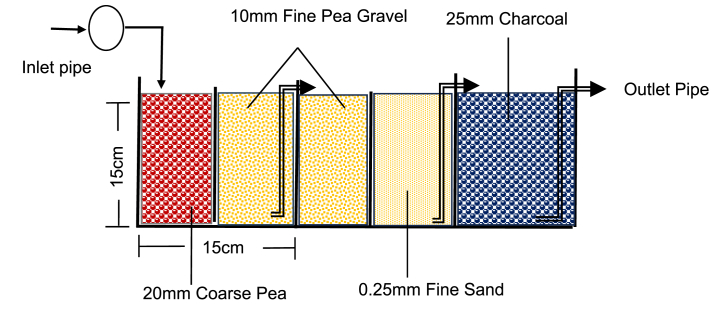
Treatment Design for the augmented sand filtration system.

An augmented sand bed treatment was designed to treat FS run-off and CW wastewater. The combination of 20 mm coarse pea gravel, 10 mm fine pea gravel, 0.25 mm fine sand (obtained from the seacoast in Cape Coast, Ghana), and 25 mm charcoal was used in the treatment. The media was thoroughly washed, dried, and sieved to acquire the media size required. The therapy comprised a horizontal tank with three chambers connected by 2-inch PVC pipes. Small-hole nets were installed in the pipe to prevent treatment materials from escaping the system. Each chamber was 15 cm by 15 cm × 15 cm in size, with a total capacity of 3.375 dm^3^ of water (Fig. 2). To condition the treatment system, fresh deionized water was passed through the bed at a flow rate of 0.125 L/min for 3 h. After drying out, aggregate samples were run through the system at the same flowrate. The composite FSR was passed through the augmented sand filter treatment media and the effluent (filtrate) was collected for analysis.

### 2.4 Physico-chemical analysis of the FS run-of and CW wastewater

Five physicochemical parameters namely pH, turbidity, total dissolved solids (TDS), electrical conductivity (EC), and salinity were analyzed in the FSR and CWW samples collected and analyzed following the manufacturer's guide after calibration. The pH of each sample was determined using the OHAUS Starter 3100C pH meter. The electrode of the pH meter was rinsed with distilled water, wiped with tissue, and calibrated with a buffer solution of pH 7.00, which is close to the expected pH of water samples. This rinsing of the probe with distilled water was repeated before each sample was evaluated. The OHAUS Starter 3100C conductivity meter was used to determine the EC, TDS, and salinity. The meter was calibrated with 12.88 μS/cm standard solution and the conductivity sensor was placed in each sample to take the measurement for the EC. The same process was repeated for the TDS and salinity. For the determination of the turbidity, the VELP Scientifica TB1 Turbidimeter was used. Before use, the meter was calibrated with 20 and 0.2 NTU standard calibration solutions. The sample vial was cleansed with distilled water and rinsed with an aliquot of the sample each time before measuring the turbidity of the sample.

### 2.5 Sample preparation and analysis of heavy metals

Samples were stored in the refrigerator prior to analysis. Wet decomposition acid digestion was performed using oxidizing agents and mineral acids to effect sample dissolution [47]. 10 ml of concentrated nitric acid (HNO_3_) was added to 50 ml of FSR and CWW samples and boiled on a hot plate until a clear solution was obtained. After 2 h, samples were removed from hot plate and 10 ml 1:1 ratio of concentrated nitrate and perchloric acid (HNO_3_–HClO_4_) was added. This allowed HClO_4_ to react explosively with organic compounds and for HNO_3_ to release metals from other materials in the sample to form highly soluble nitrate salts [48]. The mixture was then cooled to room temperature, and subsequently filtered into a volumetric flask. The mixture was finally diluted with distilled water to the 50 ml mark. For the detection of heavy metals (Cr, Cu, Ni, Pb, and Zn), the Agilent 4210 Microwave Plasma Atomic Emission Spectrometry (MP-AES) instrument at the Environmental Science Department Laboratory, Kwame Nkrumah University of Science and Technology, Kumasi Ghana was used after calibration and optimization using four multi-standard solutions containing Pb, Cd, Co, Ni, Mn, Cu, Zn, and Fe at 0.01, 0.1, 1 and 10 ppm of each heavy metal.

### 2.6 Data analysis

Descriptive statistics was performed using Microsoft Excel version xx to compute the mean and standard deviation (SD) of the physico-chemical parameters and heavy metals in the FSR and CWW, and the effluent samples after the experimental treatment of the FS run-off and CW wastewater. An independent sample *t*-test at 5% level of significance was carried out using SPSS Version 21 to determine if the mean concentrations of heavy metals in FS run-off and CW wastewater were statistically different. Pearson correlation matrix was used to analyze the relationship between heavy metals concentrations in CWW and FSR.

### 2.7 Ecological risk assessment

Due to the potential for synergistic effects of heavy metals in the environment [49], relying solely on single element indices can be inadequate for assessing contamination and associated risks. Therefore, the environmental indicators used to evaluate the risk of pollution from CW wastewater and FS run-off to the environment included single-element risk indicators such as the contamination factors and multi-element indices such as the modified degree of contamination (mCD) and the potential ecological risk index (PERI) to quantify the overall synergistic ecological risk [50].

#### 2.7.1 Contamination factor

The contamination factor (CF) is determined by dividing the metal concentrations of affected areas (C_metal_) by the background concentrations (C_ref_) [39]. The CF accounts for the contamination of a single element at a site [46] and it calculated using the equation[39].

Equation 2 $$CF=\frac{C_{metal}}{C_{ref}}$$

where CF is the contamination factor/pollution index of an individual heavy metal, C_metal_ is the metal concentration at each station, and $C_{ref}$ is the reference value (recommended limit) for each heavy metal in wastewater according to equation (2). The $C_{ref}$ are the recommended limits of wastewater heavy metals proposed by different organizations [39]. The $C_{ref}$ used in this study are the WHO [48,51,52], limits which are 0.05 for Cr, 1 for Cu, 0.02 for Ni, 0.01 for Pb, and 2 for Zn, all in mg/L.

#### 2.7.2 Ecological risk factor

The ecological risk factor, which is also the potential ecological risk (PER) [53] is given as follows[39].

Equation 3 $$\mathrm{ERF}=CF\times T_{i}$$

ERF is the ecological risk factor and $T_{i}$ is the toxic response factor/potential ecological risk coefficient. $T_{i}$ for each metal is as follows: Cr = 2, Zn = 1 and Cu, Pb and Ni = 5 [53,54]. The heavy metal contamination factor and ecological risk factor were categorized according to Refs. [46,51] as in Table 1.

**Table 1:** Categories of pollution and ecological risk levels from single and multi-element indices.

Categories	CF	ERF/PER	mCd	PERI
**0**	<1: Low	ERF<40 Low	<1.5 Unpolluted	PERI <110 Low ecological risk
**1**	1 ≤ CF < 3 Moderate	40 ≤ ERF<80 Moderate	1.5 ≤ Cd < 2 Slightly polluted	110≤ PERI <200 Moderate ecological risk
**2**	3 ≤ CF < 6 Considerate	80 ≤ ERF<160 Considerate	2 ≤ Cd < 4 Moderately polluted	200≤ PERI <300 Considerate ecological risk
**3**	CF ≥ 6 High	160 ≤ ERF<320 High	4 ≤ Cd < 8 Moderate-heavily polluted	PERI >400 Severe ecological risk
**4**		ERF≥320 Very high	8 ≤ Cd < 16 Heavily polluted	
**5**			16 ≤ Cd < 32 Severely polluted	
**References**	[46,51]	[51]	[28]	[51,53]

#### 2.7.3 Modified degree of contamination

The modified degree of contamination (mCd) is one of the significant matrices deployed for a rapid and effective evaluation of the extent of contamination at a specific location [26]. The mCd given by equation (4) was used to assess the synergistic impact of contamination of the five heavy metals [55].

Equation 4 $$mCd=\frac{1}{n}\sum_{i=1}^{n}CF$$

#### 2.7.4 Potential ecological risk index

Potential ecological risk (PERI) [41] was used to evaluate the synergistic risk of the selected metals [14]. The PERI is given as[53].

Equation 5 $$PERI=\sum_{i=1}^{n}{\mathrm{ERF}}_{n}$$

Multi-element ecological risk indices, mCd and PERI, were ranked in according to the evaluation parameters provided in Table 1**.**

### 2.8 Wastewater and run-off treatment efficacy

The removal efficiency of the raw material filter media was determined for both the physicochemical parameters and heavy metals in the run-off samples according to reported by [56].

Equation 6 $$\%\;Removal=\frac{C_{i}-C_{f}}{C_{f}}\times 100$$

Where mean $C_{i}$ mean initial parameter concentration (before treatment) and $C_{f}$ mean final parameter level (after treatment.

## 3 Results and discussion

### 3.1 Heavy metal concentrations in fuel station run-off and carwash wastewater

The sum of heavy metal concentrations obtained in the CWW were 1.59 mg/L for Cr, 4.27 mg/L for Cu, 0.52 mg/L for Ni, 25.18 mg/L for Pb, and 11.67 mg/L for Zn. FSR total heavy metal were 1.19 mg/L (Cr), 2.09 (Cu), 3.55 (Ni), 8.19 (Pb), and 3.77 (Zn). A comparison of the concentrations of heavy metals in the FS run-off to the CW wastewater shows no significant difference for chromium, copper, and zinc but statistically varied concentrations for nickel (p = 0.044) and lead (p = 0.002). This suggests that the source of chromium, copper and zinc into the run-off and the FS and the CW wastewater may be vehicular parts wearing, but the sources of lead and nickel differ at the two locations [57]. Lead and nickel concentrations in FS run-off and CW wastewater may be because carwashes and filling stations have distinct activities and therefore different heavy metal contamination sources. Fuel spills on the forecourt of filling stations [15,58] may introduce lead into run-off if leaded fuels are sold, while cleansing detergents and other acid used at CW bays may the contributing different heavy metals [59].

The mean heavy metal concentration for Cr, Cu, Ni, Pb, and Zn found in the CWW were 0.07 mg/L, 0.02 mg/L, 0.02 mg/L, 1.16 mg/L and 0.58 mg/L respectively. These results are similar to previous report [60] wherein laundry detergents were found to be sources of heavy metal pollution in wastewater. The mean heavy metal levels in CW wastewater and FS run-off compared with the WHO, US EPA and Ghana EPA recommended limits in wastewater (Table 2) showed that Cr, Ni and Pb were above the limits.

**Table 2:** Heavy metal concentration in Carwash wastewater and fuel station run-off.

Study sites	Cr (mg/L)	Cu (mg/L)	Ni (mg/L)	Pb (mg/L)	Zn (mg/L)
**CW1**	0.05 ± 0.04	0.1 ± 0.03	ND	1.95 ± 0.05	0.96 ± 0.89
**CW2**	0.02 ± 0	0.07 ± 0.02	0.01 ± 0	1.55 ± 0.15	0.09 ± 0.02
**CW3**	0.05 ± 0.03	0.12 ± 0.04	0.03 ± 0	1.58 ± 0.16	0.27 ± 0.12
**CW4**	0.03 ± 0.01	0.07 ± 0.02	0.01 ± 0	2.09 ± 0.42	0.03 ± 0.03
**CW5**	0.07 ± 0.07	0.2 ± 0.18	0.02 ± 0	1.06 ± 0.01	0.23 ± 0.21
**CW6**	0.17 ± ND	0.21 ± 0.03	0.12 ± 0.1	0.45 ± 0.2	0.95 ± 0.58
**CW7**	0.2 ± 0.12	0.14 ± 0	0.02 ± 0	0.44 ± 0.09	2.87 ± 3.25
**CW8**	ND	ND	ND	0.64 ± 0.07	0.09 ± 0
**CW9**	ND	ND	ND	0.52 ± 0.04	ND
**CW10**	0.16 ± 0.23	1.1 ± 1.54	0.01 ± 0.01	1.29 ± 1.47	0.3 ± 0.42
**Mean**	**0.07 ± 0.07**	**0.2 ± 0.19**	**0.02 ± 0.03**	**1.16 ± 0.43**	**0.58 ± 0.99**
**FS1**	0.14 ± 0.07	0.2 ± 0.14	0.05 ± 0.03	0.35 ± 0.06	0.62 ± 0.11
**FS2**	0.01 ± 0	0.01 ± 0	0.16 ± 0.14	0.31 ± 0	0.02 ± 0.01
**FS3**	0.14 ± 0	0.17 ± 0.09	0.06 ± 0.05	0.3 ± 0.01	0.54 ± 0.32
**FS4**	0.01 ± 0	0.02 ± 0	0.63 ± 0.64	0.27 ± 0.03	0.12 ± 0.02
**FS5**	0.01 ± 0	0.01 ± 0	0.02 ± 0.0	0.26 ± 0	0.03 ± 0.03
**FS6**	0.03 ± 0	0.02 ± 0	0.38 ± 0.3	0.29 ± 0	0.05 ± 0.01
**FS7**	ND	ND	ND	0.25 ± 0	ND
**FS8**	0.03 ± 0	0.04 ± 0	0.3 ± 0.42	0.28 ± 0.01	0.09 ± 0.02
**FS9**	0.17 ± 0.02	0.04 ± 0.02	0.08 ± 0.08	0.5 ± 0.23	0.35 ± 0.44
**FS10**	0.01 ± 0.01	0.42 ± 0.53	0.05 ± 0.06	0.26 ± 0.26	0.03 ± 0.03
**Mean**	**0.05 ± 0.02**	**0.09 ± 0.16**	**0.17 ± 0.21**	**0.31 ± 0.1**	**0.18 ± 0.15**
**P values**	**0.523**	**0.343**	**0.044**	**0.002**	**0.200**
**WHO**^**a**^**limits**	0.05	1	0.02	0.01	2
**US EPA**^**b**^	0.05	–	0.2	0.006	
[53]	<1	0.94–3.8	–	<1	1.15–3.0
[54]	0.42	0.06	–	0.28	0.18

a = [[29], [30], [31]]; b = [52].

**Fig. 3:**
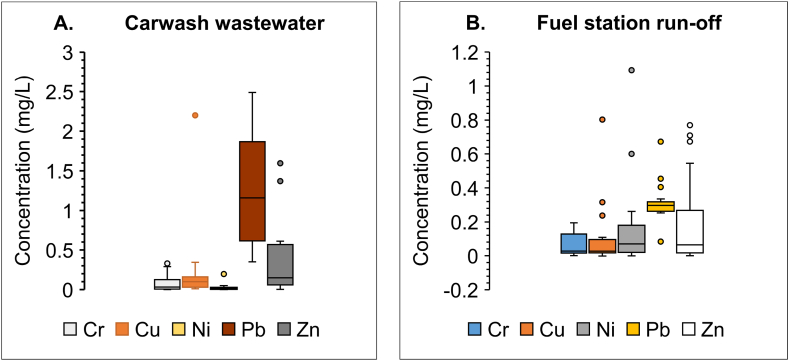
Concentrations of heavy metals [A] in carwash wastewater and [B] in fuel station run-off.

Table 2 shows that CWW chromium level in this study agrees with other studies [61,62], while Cu and Zn were higher than findings reported by Ref. [62] but less than the value recorded by Ref. [61]. These differences suggest that Cu and Zn may originate from the dust in the area which depends on geology, hence the difference in the study from Northern and Ashant regions of Ghana. The similarity in Cr levels suggest that this metal is emanating from vehicular worn parts. The Pb concentrations in CWW reported in this study were higher than background levels, which shows that Pb from detergents, lubricants or other chemicals are entering the CWW. The average concentration of heavy metals in CWW varied between non-detectable concentrations and 2.48 mg/L (Fig. 3, panel A). The order of heavy metal concentrations in the CWW is Pb > Zn > Cu > Cr > Ni but in the FSR the order was Pb > Ni > Zn > Cr > Cu (Fig. 3, panel B).

The dataset includes measurements of chromium concentration ranging from ND to 0.3 mg/L, copper had a median concentration of 0.1 mg/L and varied between 0.01 mg/L and 0.3 mg/L. The concentration of nickel exhibits a consistently lower range, with values ranging from ND to 0.05 mg/L whilst zinc in the samples varied between 0.03 mg/L and 0.61 mg/L, with a median value of 0.14 mg/L. The recorded values of Pb ranged from a minimum of 0.35 mg/L to a maximum of 2.48 mg/L with a median concentration of 1.15 mg/L, which is higher than the WHO limits [41,44,63].

The concentrations of Cr, Cu, Ni, Pb, Zn in FSR shows that nickel had the highest concentration and range from below detection limit to 1.0932 mg/L whilst Cr exhibited the lowest overall concentration, with all recorded values consistently at 0.195 mg/L. Cu, Pb, and Zn displayed relatively moderate concentration ranges, highlighting the variability within their measurements.

The diverse ranges and extremities of the dataset, emphasize the need for further investigation into potential sources, influences, and implications of these heavy metal concentrations from CWW and FSR. The mean values of Pb and Cr in FSR, 0.31 mg/L and 0.05 mg/L respectively (Table 2) are higher than the values reported by Ref. [64] whilst the mean concentration of FSR nickel in this study (0.17 mg/L) is found to be higher than that of [64].

3.2 Physicochemical qualities of carwash wastewater and fuel station run-off.

Table 3 shows the mean and standard deviation (Mean ± SD), and p-values of each physicochemical parameter analyzed in CWW and FSR. The pH, EC, and TDS values were within the Ghana EPA and WHO limits, except for turbidity which was about 94 times above the limits. The pH levels in CWW ranged from 6.74 to 8.11 and from 6.80 to 8.40 for FSR, with a mean pH concentration of 7.48. The pH values are above the pH range of rainwater reported by Ref. [65] in Kumasi metropolitan area. The increase in pH of the FSR and CWW suggest that rainwater falling on the fuel station forecourt mixes with alkaline substances. The CWW pH may be due to the use of bleach and other alkaline cleaning products. The turbidity measurement in CWW were significantly higher than that of FSR (p = 0.79) and it ranged from 2.37 to 205 NTU and from 1.49 to 187 in FSR, which shows that CWW turbidity levels are slightly higher than FSR turbidity. This is because the fuel stations have paved or tarred forecourt in contrast to CW bays, and most cars are going for a wash are muddy as result of driving on untarred roads and through rain-filled potholes during the season of sampling. The mean turbidity of CWW was 169.61 NTU which exceeded the Ghana EPA standard value [66] by 56%. The results also reveal that CWW TDS varied from 168 to 369 mg/L, whereas that of FSR was 30.3–356 mg/L, with a mean TDS in CWW of 320 mg/L and that of FSR was 172.83 mg/L, both within WHO and Ghana EPA guidelines. Standard deviation of 73.17 for CWW and 133.51 for FSR reveal high turbidity variability across carwashes since some are patched and others are not. The mean TDS in CWW was substantially greater than and significantly different (p-value of 0.04) from that of FSR and is due to the addition of soluble cleaning chemicals during the vehicle washing process. The CW wastewater EC values range from 338 to 734 μS/cm and FFSs runoff was 60.5–571 μS/cm. Although the mean EC concentration in CWW met Ghana EPA limit, it varied substantially. This may be due to the differences in detergents and chemicals used in the cleaning of the vehicles. FSR had a mean of 272.55, but an SD of 266.53 shows significant EC variability, which may be attributed to quality differences of fuel sold at filling station [67]. There was a significant difference between the EC of CW wastewater and that of FS run = off (p = 0.01). Salinity values range from 0.16 to 0. 36 PSU for CWW and 0.03 to 0.35 PSU for FSR.

**Table 3:** Comparison of physicochemical parameters in CW wastewater and FS run-off.

Samples	pH	Turbidity (NTU)	TDS (mg/L)	EC (μS/cm)	Salinity (PSU)
CW wastewater	7.48 ± 0.05	169.6 ± 19	320.0 ± 73	640.7 ± 145	0.31 ± 0.07
FS run-off	7.47 ± 0.11	142.14 ± 1.6	172.8 ± 134	272.55 ± 266.53	0.17 ± 0.12
**P-values**	**0.95**	**0.79**	**0.04**	**0.01**	**0.06**

### 3.2 Correlation of heavy metal levels in fuel station fun-off and carwash wastewater

Table 4 shows the inter-elemental correlations within the heavy metal levels of FSR and CWW which provide a comprehensive knowledge of the relationships within both environmental systems. Strong positive correlations observed were Cr and Pb in FSR (0.8), Cr and Zn in FSR (0.9) and between CWW and FSR copper concentrations (0.8) as well as between Zn concentrations in CWW (0.7). These results suggest that copper and zinc in FSR and CWW may originate from the same source. Similarly, correlations of lead, zinc and chromium in topsoil samples from the Kumasi metropolis has been reported [68]. This that dust from topsoil is the source of these metals and may explain the correlation between Cr and Pb, and Cr and Zn in the FSR.

**Table 4:** Inter-elemental correlation of heavy metal levels in FSR and CWW.

Metals	Cr (CW)	Cr (FS)	Cu (CW)	Cu (FS)	Ni (CW)	Ni (FS)	Pb (CW)	Pb (FS)	Zn (CW)	Zn (FS)
Cr (CW)	**1.0**									
Cr (FS)	−0.5	**1.0**								
Cu (CW)	**0.6**	−0.3	**1.0**							
Cu (FS)	0.2	0.2	**0.8**	**1.0**						
Ni (CW)	**0.5**	−0.2	0.0	−0.2	**1.0**					
Ni (FS)	−0.2	−0.3	−0.2	−0.3	0.3	**1.0**				
Pb (CW)	−0.3	0.1	0.1	0.3	−0.3	0.2	**1.0**			
Pb (FS)	−0.5	**0.8**	−0.3	0.0	−0.3	−0.2	−0.1	**1.0**		
Zn (CW)	**0.7**	−0.2	0.0	−0.2	0.2	−0.3	−0.4	−0.3	**1.0**	
Zn (FS)	−0.4	**0.9**	−0.3	0.3	−0.2	−0.2	0.4	**0.5**	−0.1	**1.0**

FS = Fuel station; CW = Carwash Significance level: P-value = 0.05.

### 3.3 Ecological risk assessment

From the single-element ecological risk indicators, CF and ERF, considerable variations in elemental contamination and risk levels were seen across the different heavy metals in FSR and CWW. Lead was found to pose a high level of ecological risk while chromium posed low to considerate, copper from considerate to very high, nickel varied from low to very high risk and zinc was consistently low (Table 5). Given the high toxicity of Pb [27,28] and its correspondingly low allowable limit in environmental samples [53,54], it can understandable that the observed high levels of lead in this study will pose a high ecological risk. The mCd and PERI for the heavy metals (Table 6) shows that the cumulative synergistic risks of all the heavy metals (Cr, Cu, Ni, Pb, and Zn) in CWW pose a significant risk to the ecology of receiving water bodies of the untreated wastewater. According to the mCd, CWW is extremely polluted with Pb, and the PERI showed that Pb poses severe ecological risk to aquatic species in the receiving waters of the untreated wastewater. Chromium, copper, nickel, and zinc contaminations are low to moderate and exhibited low ecological risk in the PERI index. The single-element contamination factor (CF) and ecological risk factor (ERF) for heavy metal in the FSR. There is a variation in contamination and risk levels across different metals and FSs. Chromium and zinc showed consistently low to moderate contamination, while copper was consistently low. Contamination of nickel varies from low to very high, whilst lead consistently shows high contamination and high-risk levels across all stations. The results suggests that lead has the highest potential to cause harm to the ecosystem, among the metals studied. The modified degree of contamination (mCD) and the potential ecological risk index (PERI) for the heavy metals in FS run-off showed that nickel and lead had extreme and severe degrees of contamination respectively; and posed severe ecological risks in the PERI index, while Cr, Cu, and Zn contamination ranked as unpolluted were posed low ecological risk.

**Table 5:** Individual metal contamination factor and ecological risk factor (ERF) of fuel forecourt wastewater.

	PLI & Rank	Heavy metals					Car wash	PLI & Rank	Heavy metals				
		Cr	Cu	Ni	Pb	Zn			Cr	Cu	Ni	Pb	Zn
**FS1**	CF [Risk]	2.8 [M]	0.2 [L]	2.6 [M]	35.6 [VH]	0.3 [L]	**CW1**	CF [Risk]	1.2 [M]	0.1 [L]	0.4 [L]	195.3 [VH]	0.5 [L]
	ERF [Risk]	5.7 [L]	1.1 [L]	12.8 [L]	178.2 [H]	0.6 [L]		**ERF** [Risk]	2.3 [L]	0.5 [L]	2.1 [L]	976.6 [VH]	1 [L]
**FS2**	CF [Risk]	0.3 [L]	ND [L]	8.1 [VH]	31.6 [VH]	ND [L]	**CW2**	CF [Risk]	0.6 [L]	0.1 [L]	0.8 [L]	155.1 [VH]	ND [L]
	ERF [Risk]	0.5 [L]	0.1 [L]	40.6 [M]	157.8 [C]	ND [L]		**ERF** [Risk]	1.1 [L]	0.4 [L]	4 [L]	775.6 [VH]	0.1 [L]
**FS3**	CF [Risk]	2.9 [M]	0.2 [L]	3.4 [C]	30.8 [VH]	0.3 [L]	**CW3**	CF [Risk]	1.2 [M]	0.1 [L]	1.7 [M]	158.9 [VH]	0.1 [L]
	ERF [Risk]	5.7 [L]	0.9 [L]	17 [L]	153.8 [C]	0.5 [L]		**ERF** [Risk]	2.3 [L]	0.6 [L]	8.3 [L]	794.6 [VH]	0.3 [L]
**FS4**	CF [Risk]	0.4 [L]	ND [L]	31.9 [VH]	27.6 [VH]	0.1 [L]	**CW4**	CF [Risk]	0.7 [L]	0.1 [L]	0.7 [L]	209.2 [VH]	ND [L]
	ERF [Risk]	0.8 [L]	0.1 [L]	159.7 [C]	137.9 [C]	0.1 [L]		**ERF** [Risk]	1.5 [L]	0.3 [L]	3.3 [L]	1045.9 [VH]	ND [L]
**FS5**	CF [Risk]	0.3 [L]	0.1 [L]	1 [M]	26.6 [VH]	ND [L]	**CW5**	CF [Risk]	1.6 [M]	0.5 [L]	1.1 [M]	106.3 [VH]	0.1 [L]
	ERF [Risk]	0.7 [L]	0.1 [L]	5.1 [L]	132.8 [C]	ND [L]		**ERF** [Risk]	3.2 [L]	1.1 [L]	5.7 [L]	531.5 [VH]	0.2 [L]
**FS6**	CF [Risk]	0.6 [L]	0.1 [L]	19.1 [VH]	29 [VH]	ND [L]	**CW6**	CF [Risk]	3.4 [C]	0.2 [L]	6.1 [VH]	46 [VH]	0.5 [L]
	ERF [Risk]	1.3 [L]	0.1 [L]	95.4 [C]	145.1 [C]	0.1 [L]		**ERF** [Risk]	6.8 [L]	1.1 [L]	30.5 [L]	229.8 [H]	1 [L]
**FS7**	CF [Risk]	ND [L]	ND [L]	ND [L]	25.3 [VH]	ND [L]	**CW7**	CF [Risk]	4 [C]	0.1 [L]	1.1 [M]	44.1 [VH]	1.4 [M]
	ERF [Risk]	ND [L]	ND [L]	0.1 [L]	126.6 [C]	ND [L]		**ERF** [Risk]	8 [L]	0.7 [L]	5.5 [L]	220.4 [H]	2.9 [L]
**FS8**	CF [Risk]	0.8 [L]	ND [L]	15.1 [VH]	28.7 [VH]	ND [L]	**CW8**	CF [Risk]	ND [L]	ND [L]	ND [L]	64.9 [VH]	ND [L]
	ERF [Risk]	1.5 [L]	0.2 [L]	75.3 [M]	143.7 [C]	0.1 [L]		**ERF** [Risk]	0.1 [L]	ND [L]	0.1 [L]	324.7 [VH]	0.1 [L]
**FS9**	CF [Risk]	3.5 [C]	0.1 [L]	4.4 [C]	50.4 [VH]	0.2 [L]	**CW9**	CF [Risk]	ND [L]	ND [L]	−0.1 [L]	52.6 [VH]	ND [L]
	ERF [Risk]	7 [L]	0.3 [L]	22.1 [L]	252.1 [H]	0.4 [L]		**ERF** [Risk]	0.1 [L]	ND [L]	−0.3 [L]	262.9 [H]	ND [L]
**FS10**	CF [Risk]	0.4 [L]	0.4 [L]	2.6 [M]	26.9[VH]	ND [L]	**CW10**	CF [Risk]	3.3 [C]	1.1 [M]	0.5 [L]	129.4 [VH]	0.2 [L]
	ERF [Risk]	0.7 [L]	2.1 [L]	13.1 [L]	134.3 [C]	ND [L]		**ERF** [Risk]	6.6 L]	5.5 [L]	2.5 [L]	647.2 [VH]	0.3 [L]

FS = Fuels station; CW = Car wash service station; CF = contamination factor; ERF = Environmental Risk factor; L = low; M = medium; C = considerable; H = high; VH = very high.

**Table 6:** Ecological risk assessment (mCd and PERI) of heavy metals from FS run-off and CW wastewater.

	Eco. Risk assessments	Heavy metals				
		Cr	Cu	Ni	Pb	Zn
**Fuel station run-off**	mCd Values	2.39	0.19	17.65	62.49	0.19
	Contamination Degree	Moderately polluted	Unpolluted	Severely polluted	Extremely Polluted	Unpolluted
	PERI values	23.87	4.87	441.16	1562.15	1.89
	Ecological Risk Index	Low	Low	Severe	Severe	Low
**Carwash wastewater**	mCd values	3.20	0.41	2.47	232.37	0.58
	Contamination Degree	Moderately polluted	Unpolluted	Moderately polluted	Extremely Polluted	Unpolluted
	PERI values	31.97	10.32	61.66	5809.28	5.83
	Ecological Risk Index	Low	Low	Low	Severe	Low
**Treated FSR effluent**	mCD values	0.32	0.022	1	0.8	0.014
	Contamination Degree	Unpolluted	Unpolluted	Unpolluted	Unpolluted	Unpolluted
	PERI values	3.2	0.55	25.00	20.00	0.14
	Ecological Risk index	Low	Low	Low	Low	Low
**Treated CWW effluent**	mCD values	0.44	0.01	0.40	1.00	0.01
	Degree of Contamination	Unpolluted	Unpolluted	Unpolluted	Unpolluted	Unpolluted
	PERI	4.40	0.30	10.00	25.00	0.12
	Ecological Risk index	Low	Low	Low	Low	Low

### 3.4 Efficacy of the charcoal-augmented sand filter treatment of CWW and FSR

**Fig. 4:**
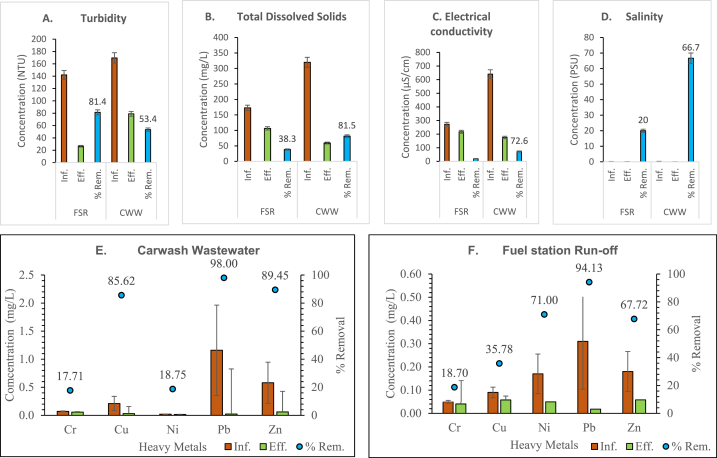
Concentrations and removal efficiency of the augmented sand filtration system for [A] Turbidity, [B] Total suspended solids, [C] Electrical conductivity, [D] Salinity, [E] heavy metals in CWW and [F] heavy metals in FSR.

The treatment system removed turbidity (81%), TDS (38%), and EC (20%) in FSR and significantly reduced turbidity in the CWW by 53%, TDS by 82%, and EC and salinity by 82% each (Fig. 4, panels A to D). The removal percentages were high in CWW compared with performance in FSR, this may be due to the highly turbid nature of the CWW which make the sand filter bed most suited to trap the suspended particles in the wastewater. The use of gravel, sand, and charcoal beds for the treatment of water and wastewater in different treatment set-ups have reported good outcomes. The removals are similar to other findings where chitosan-sand and charcoal-sand as filter materials significantly reduced turbidity, TDS, and EC in river water by 94, 85 and 85% respectively [69].

The heavy metal removal efficiencies in the CWW (Fig. 4, panel E) and FSR (Fig. 4, panel F) reveal varying degrees of success for each metal. Lead, copper and zinc in CWW demonstrated high removal efficiencies of 98%, 86% and 89% respectively, while Cr and Ni recorded low reductions of 18% and 19% respectively. The results for Pb and Zn are close to the average efficiencies reported by Ref. [69] using chitosan-sand and chitosan-charcoal filter media. This suggest that Pb, Cu and Zn may be in the insoluble form and hence, trapped in the sand-filter along with the turbidity particle, or might have been adsorbed by the charcoal, if it existed as soluble complexes. Similarly, the removal efficiency of the heavy metal from the FSR showed the highest removal efficiencies for Pb (94%), Ni and (71%). Copper and zinc reported moderate removal efficiencies of 36% and 68%, respectively. Chromium (Cr) showed the lowest removal efficiency among the studied metals, with a removal rate of 19%.

## 4 Conclusion and recommendation

Anthropogenic activities at fuel station and carwash premises are contributing to Cr, Cu, NI, Pb and Zn contamination of surface and groundwater through the discharge of FSR and CWW. The overall heavy metal concentrations in CWW wastewater were 1.5904 mg/L Cr, 4.2739 mg/L Cu, 0.5173 mg/L Ni, 25.177 mg/L Pb, and 11.667 mg/L Zn. FSR total heavy metals were 1.1855 mg/L Cr, 2.0946 mg/L Cu, 3.5533 mg/L Ni, 8.1886 mg/L Pb, and 3.7701 mg/L Zn. Although the CWW and FSR pH, EC, and TDS were within Ghana EPA and WHO guidelines, the turbidity significantly higher than the limits and therefore the FSR and CWW should be intercepted and treated before discharge. The Cr, Cu, Ni, Pb, and Zn mean concentrations in CWW were 0.07, 0.02, 0.02 mg, 1.16, and 0.58 mg/L. Cr, Ni, and Pb exceeded WHO and Ghana EPA standards whilst Pb in CWW had greater levels than background concentrations, showing that the car washing activity is contamination the receiving waters with Pb. Furthermore, the was a strong correlation between and with CWW and FSR samples for Cu, Zn, and Cr suggests a similar source for the enrichment, possibly dust from the surrounding topsoil or wearing vehicle brake systems or from fuel and lubricants spills at the two premises. Pb contents in CWW and FSR presented a significant ecological concern (PERI = 1562.15). Although low amounts of Cr, Cu, Ni, and Zn were found, the continuous release of CWW and FSR effluent into adjacent streams may affect ecosystems and hence, FSR and CWW should be impounded and treated. The augmented sand filter treatment achieved high removals of Pb and Zn from CWW and FSR in comparison with the others Cr, Cu and Ni.

These findings necessitate the following recommendations: additional heavy metals in CWW and FSR and their ecological risks should be studied. Future research should investigate the mechanism by which the charcoal-augmented sand filter system removes the heavy metal in CWW and FSR, and the health risks posed by heavy-metal-contaminated FSR and CWW. Regulatory agencies should consider the use of charcoal-augmented sand filtration system as the minimum requirement or best practice recommendations for the treatment of FSR and CWW, and instructional initiatives should all be put in place in policies governing the construction and operation of fuels stations and carwash facilities.

## CRediT authorship contribution statement

**Emmanuel Leekplah Cuput:** Writing – original draft, Investigation, Funding acquisition. **Lawson Mensah:** Writing – review & editing, Visualization, Conceptualization. **Ethel Bentil:** Investigation, Data curation. **Victoria Amponsah:** Resources, Methodology. **Bright Kwaku Agbekey:** Supervision, Project administration.

## Declaration of competing interest

The authors declare that they have no known competing financial interests or personal relationships that could have appeared to influence the work reported in this paper.
